# Lower eGFR is independently associated with embolization‑requiring severe bleeding after percutaneous renal biopsy: a matched case–control study with dose–respons e analysis

**DOI:** 10.1080/0886022X.2026.2714663

**Published:** 2026-09-03

**Authors:** YiFei Xu, Weidong Huang, Xuelian Wu, Honghua Lv, Qiankun Zhang, Wenhui Lei

**Affiliations:** ^a^ Department of Rheumatology, The Fifth Affiliated Hospital of Wenzhou Medical University, Lishui, China; ^b^ Nephrology and Urology Center, The Fifth Affiliated Hospital of Wenzhou Medical University, Lishui, China; ^c^ Department of Nephrology, The Fifth Affiliated Hospital of Wenzhou Medical University, Lishui, China

**Keywords:** Estimated glomerular filtration rate, percutaneous renal biopsy, severe bleeding, renal artery embolization

## Abstract

**Background:**

Reduced estimated glomerular filtration rate (eGFR) is a suspected risk factor for bleeding after percutaneous renal biopsy, but its association with hemorrhage requiring embolization is unclear. We evaluated this association using a matched case‑control study.

**Methods:**

This single‑center, retrospective, matched case‑control study included patients who underwent ultrasound‑guided native renal biopsy between 2012 and 2025 at a Chinese tertiary hospital. Cases (*n* = 16) were patients with severe bleeding requiring selective renal artery embolization; controls (*n* = 64) had no post‑biopsy bleeding, matched 1:4 by sex and age. eGFR was calculated using the CKD‑EPI equation, analyzed as a continuous variable and categorized according to KDIGO criteria (<30, 30–60, ≥60 mL/min/1.73 m^2^). Multivariable logistic regression, restricted cubic spline analysis, subgroup analyses, LASSO regression, and sensitivity analyses (ROC curve, dichotomized eGFR, complete‑case analysis) were performed.

**Results:**

In fully adjusted models, each 1 mL/min/1.73m^2^ increase in eGFR was associated with 4% lower bleeding risk (OR 0.96, 95% CI 0.93–0.98, *p* = 0.007). eGFR ≥60 versus <30 mL/min/1.73 m^2^ gave OR 0.09 (95% CI 0.01–0.75, *p* = 0.03), with a significant dose‑response trend (P for trend = 0.02). Restricted cubic spline analysis revealed a linear inverse association. Results were consistent across subgroups and sensitivity analyses.

**Conclusions:**

Lower eGFR may be independently associated with increased risk of embolization-requiring bleeding after renal biopsy in a linear dose-response manner. Given the limited sample size, these exploratory findings require validation in larger, prospective cohorts. eGFR may aid in preoperative risk stratification.

## 1. Introduction

Percutaneous renal biopsy is the gold standard for diagnosing renal parenchymal disease, providing essential information for pathological classification, treatment decisions, and prognosis [1]. With advances in ultrasound guidance and biopsy devices, the safety of this procedure has improved significantly [2]. However, as an invasive procedure, post-biopsy bleeding remains the most common complication. Although most bleeding manifests as asymptomatic perirenal hematomas, severe bleeding events can lead to significant hemoglobin decline, require blood transfusion, or necessitate selective renal artery embolization-thereby prolonging hospitalization, increasing healthcare burden, and potentially endangering life [3,4]. Therefore, preoperative identification of patients at high risk for bleeding, particularly those prone to severe bleeding, remains a clinical priority.

Previous studies have identified various risk factors for post-biopsy bleeding, including female sex, advanced age, hypertension, impaired kidney function (e.g. lower estimated glomerular filtration rate [eGFR], elevated serum creatinine or urea nitrogen), thrombocytopenia, coagulation abnormalities, and number of needle passes [5–8]. Although post‑biopsy hematomas are commonly detected on ultrasound, their clinical significance varies widely, and there is no standardized classification system distinguishing perirenal from subcapsular hematomas in the literature. Reported incidence rates of post‑biopsy hematomas range from 5% to 85% depending on the imaging modality used, timing of assessment, and definition applied [9]. However, most hematomas are small, clinically silent, and resolve spontaneously without intervention. The lack of consensus on hematoma grading and the heterogeneity in outcome definitions across studies have made it difficult to compare bleeding rates between cohorts and to establish consistent risk prediction models. Therefore, focusing on the most clinically meaningful and objective endpoint—bleeding requiring selective renal artery embolization—avoids the ambiguity inherent in variable definitions of hematoma severity and ensures a more robust assessment of clinically significant hemorrhage.Among these, eGFR—a core indicator of kidney function—has garnered considerable attention in recent years. Multiple studies suggest that kidney impairment is an independent risk factor for post-biopsy bleeding. A prospective multicenter study by Halimi et al. [10] showed that each 10 mL/min/1.73 m^2^ decrease in eGFR was associated with an 18% increase in the risk of major bleeding events. A retrospective analysis of 1,146 patients similarly confirmed an independent association between lower eGFR and bleeding risk [4]. However, other studies have not observed a significant association between eGFR and bleeding [6], and one study found that after adjusting for hemoglobin, the association was no longer statistically significant [5]. These discrepancies suggest that the relationship may be influenced by study design, population characteristics, or the definition of bleeding outcomes. More importantly, most existing studies define ‘severe bleeding’ as the need for transfusion or a hemoglobin drop of ≥1 g/dL, with few specifically focusing on the most severe bleeding events requiring vascular intervention.

The mechanisms by which kidney impairment increases bleeding risk are multifaceted. The uremic environment can lead to platelet dysfunction—including abnormalities in platelet adhesion, aggregation, and release—which is a core mechanism underlying bleeding tendency in patients with renal failure [11]. In addition, anemia, endothelial dysfunction, and coagulation factor abnormalities may further increase bleeding risk [12]. Patients with end-stage kidney disease often receive concomitant antiplatelet or anticoagulant therapy, which further elevates perioperative bleeding risk. Theoretically, lower eGFR may influence hemostatic balance through these multiple pathways, thereby increasing post-biopsy bleeding risk.

Nevertheless, there is a lack of matched case–control studies specifically examining the association between eGFR and severe bleeding requiring embolization after percutaneous renal biopsy. To address this gap, we conducted a matched case–control study to evaluate the independent association between eGFR and severe bleeding requiring selective renal artery embolization following percutaneous renal biopsy, with the goal of providing a simple preoperative risk stratification tool for clinical practice.

## 2. Methods

### 2.1. Study design and population

**Figure 1.**
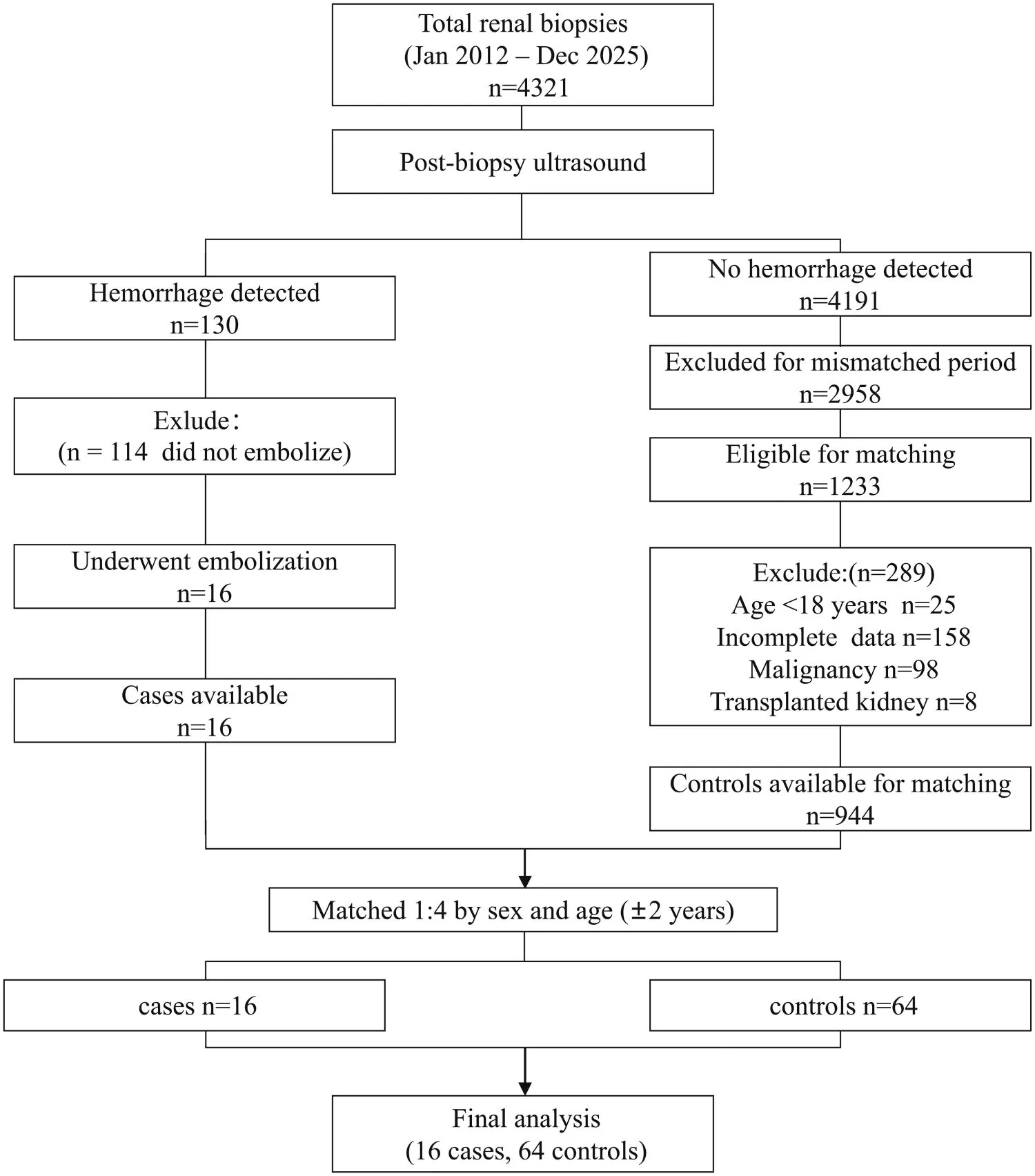
Flow diagram of participant selection. Of 4,321 patients undergoing renal biopsy, 130 had post‑biopsy hemorrhage; 16 required embolization and were included as cases. From 4,191 patients without hemorrhage, 64 controls were matched 1:4 by sex and age (±2 years) after excluding those with age <18 years, incomplete data, malignancy, or transplanted kidney. Final analysis: 16 cases, 64 controls.

This single-center, retrospective, matched case–control study was conducted in the Department of Nephrology at the Fifth Affiliated Hospital of Wenzhou Medical University (Lishui Central Hospital), a tertiary care hospital serving as a regional referral center for kidney diseases. The study population comprised patients who underwent ultrasound-guided percutaneous native renal biopsy at this institution between January 2012 and December 2025. Cases were defined as patients who developed severe post‑biopsy bleeding requiring selective renal artery embolization. Controls were patients with no evidence of bleeding on post‑biopsy ultrasound examination. Post‑biopsy ultrasound was routinely performed at 12 h after the procedure.To minimize confounding, both cases and controls were required to have no ultrasonographic signs of chronic kidney changes, such as reduced kidney size, cortical thinning, or increased cortical echogenicity. Controls were matched to cases 1:4 by sex and age (±2 years), and were selected from patients who underwent biopsy within the same period (±3 months). Exclusion criteria were: (1) age <18 years; (2) incomplete clinical or pathological data; (3) history of malignancy; or (4) transplant kidney biopsy. Each patient was included only once. After matching, 80 patients were included in the final analysis, with 16 cases and 64 controls. The participant selection process is illustrated in Figure 1.

Matching by sex and age was performed because both factors are established independent risk factors for post‑biopsy bleeding and are strongly correlated with eGFR levels. This matching strategy allowed us to effectively control for these two major confounders, thereby enabling a more precise estimation of the independent effect of eGFR on severe bleeding.

### 2.2. Bias control and ethical approval

To minimize potential bias, the following measures were implemented: (1) All biopsies were performed by a single experienced nephrologist using a 16‑gauge automated biopsy needle under real‑time ultrasound guidance, with three needle passes performed for each patient, which helped standardize the procedure and minimize operator‑related variability. (2) Data extraction was performed independently by two trained researchers who were blinded to case‑control status; any discrepancies were resolved through discussion or consultation with a third researcher. This study adhered to the principles of the Declaration of Helsinki. The study protocol was approved by the Ethics Committee of the Fifth Affiliated Hospital of Wenzhou Medical University (2026(I)-255-01). Given the retrospective design, the requirement for informed consent was waived by the Ethics Committee of the Fifth Affiliated Hospital of Wenzhou Medical University.

### 2.3. Variables and definitions

The primary outcome was severe bleeding, defined as post‑biopsy hemorrhage requiring selective renal artery embolization.The decision to perform embolization was based on any of the following categories of indications: (1) Angiographic evidence of vascular injury: active contrast extravasation on renal computed tomography angiography (CTA); (2) hemodynamic instability (systolic blood pressure persistently <90 mmHg or a decrease ≥30 mmHg from baseline that did not respond to fluid resuscitation); (3) a hemoglobin decrease ≥20 g/L within 24 h, or a continuous decline below this threshold accompanied by significant clinical symptoms (e.g. severe flank pain, abdominal distension, tachycardia); or (4) progressive expansion of a perirenal hematoma on ultrasound combined with any of the above clinical or imaging findings. All embolization procedures were performed under digital subtraction angiography guidance.

The primary exposure variable was estimated glomerular filtration rate (eGFR), calculated using the CKD-EPI equation based on serum creatinine, age, and sex. For categorical analyses, eGFR was classified into three levels according to the KDIGO clinical practice guideline: <30, 30–60, and ≥60 mL/min/1.73 m^2^. eGFR was also analyzed as a continuous variable.

All clinical and laboratory data were independently extracted from the electronic medical record system by two trained researchers blinded to case–control status. Laboratory values were obtained from the first measurement performed during hospitalization prior to the biopsy. All tests were performed using uniform methods and reagents in the same hospital laboratory. Additional variables collected included demographic characteristics, lifestyle factors (smoking and alcohol consumption), comorbidities (hypertension, diabetes mellitus, history of malignancy), medication use (antiplatelet agents, anticoagulants, lipid‑lowering drugs), and laboratory parameters (white blood cell count, hemoglobin, platelet count, prothrombin time, activated partial thromboplastin time, international normalized ratio, D‑dimer, albumin, blood urea nitrogen, serum creatinine, eGFR, 24‑hour urinary protein, triglycerides, and total cholesterol).

Pathological findings were documented for all included patients but were not included in the primary analysis, as the study was designed to evaluate routinely available pre‑biopsy clinical and laboratory parameters for risk stratification.

### 2.4. Sample size and missing data handling

Severe post‑biopsy bleeding is a rare event, and this study was exploratory in nature. We included all eligible patients with severe bleeding between 2012 and 2025; thus, no *a priori* sample size calculation was performed. Missing data were handled using multiple imputation by chained equations with five imputations. The imputation model included all covariates and the outcome variable. In sensitivity analyses, complete‑case analyses were performed to validate the robustness of the results.

### 2.5. Statistical analysis

Continuous variables were tested for normality. Normally distributed variables were presented as mean ± standard deviation and compared using the independent samples t‑test. Non‑normally distributed variables were presented as median (interquartile range) and compared using the Mann–Whitney U test. Categorical variables were presented as frequency (percentage) and compared using the chi‑square test or Fisher’s exact test.

Multivariable logistic regression was used to evaluate the independent association between eGFR and severe bleeding risk. Three sequential models were constructed: Model I adjusted for age and sex; Model II further adjusted for smoking, alcohol consumption, diabetes, and hypertension; Model III additionally adjusted for platelet count, prothrombin time, activated partial thromboplastin time, albumin, D‑dimer, triglycerides, total cholesterol, and 24‑hour urinary protein.

To explore potential non‑linear relationships, restricted cubic spline regression with four knots was applied, treating eGFR as a continuous variable.To avoid overfitting due to the number of candidate variables, LASSO (Least Absolute Shrinkage and Selection Operator) regression was employed for covariate selection. The optimal tuning parameter λ was chosen using the ‘1‑standard error’ rule (1‑SE λ) based on ten‑fold cross‑validation to balance model fit and parsimony. The LASSO model selected prothrombin time, activated partial thromboplastin time, platelet count, and 24‑hour urinary protein (dichotomized at the median of 1.55 g/24h) for inclusion in the final multivariable logistic regression models.

Subgroup analyses were performed to evaluate consistency of the association across strata of albumin level (<30 vs. ≥30 g/L), sex, diabetes, and hypertension. Odds ratios were estimated using the fully adjusted Model III, and interactions were tested by including product terms between the stratification variable and eGFR.

Sensitivity analyses included: (1) re‑classifying eGFR using the optimal cut‑off determined by receiver operating characteristic (ROC) curve analysis and repeating multivariable logistic regression; (2) assessing the predictive performance of eGFR for severe bleeding using the area under the ROC curve (AUC); and (3) conducting LASSO‑based covariate selection as described above. All statistical analyses were performed using R software (version 4.5.1). A two‑sided *p* < 0.05 was considered statistically significant.

## 3. Results

### 3.1. Baseline characteristics of the study population

A total of 80 patients who underwent percutaneous renal biopsy were included in this study, comprising 16 cases (severe post‑biopsy bleeding requiring selective renal artery embolization) and 64 controls (no clinically significant bleeding). After 1:4 matching by sex and age (±2 years), the two groups were well balanced in terms of sex and age distribution. The median age of the overall population was 54.0 years (interquartile range, 42.0–64.3 years), and 65.0% (52/80) were male. No significant differences were observed between cases and controls in age (*p* = 0.52) or sex (*p* = 0.73).

Baseline characteristics stratified by bleeding status are summarized in Table 1. Regarding lifestyle factors, the proportions of smokers and alcohol consumers did not differ significantly between the two groups (*p* = 0.38 and *p* = 0.55, respectively). Missing data were minimal: prothrombin time (PT) had 1 missing value (1.2%), activated partial thromboplastin time (APTT) had 1 missing value (1.2%), and D‑dimer had 1 missing value (1.2%); all other variables were complete.Overall, only 3 out of 2,400 data points (0.125%) were imputed.In terms of laboratory parameters, the case group exhibited worse kidney function. Blood urea nitrogen (BUN: 15.29 ± 9.21 mmol/L vs. 6.83 ± 2.46 mmol/L, *p* = 0.002) and serum creatinine (Cr: 303.62 ± 280.22 μmol/L vs. 96.78 ± 58.75 μmol/L, *p* = 0.01) were significantly higher in cases than in controls, whereas estimated glomerular filtration rate (eGFR) was significantly lower (43.82 ± 36.40 mL/min/1.73 m ^2^ vs. 82.10 ± 28.77 mL/min/1.73 m ^2^, *p* < 0.001). The 24‑hour urinary protein level was significantly lower in the case group (1.72 ± 1.30 g/24h vs. 3.50 ± 3.54 g/24h, *p* = 0.002).

**Table 1.** Baseline characteristics of patients with and without severe post-biopsy bleeding.

Variables	Control (*n* = 64)	Case (*n* = 16)	P value
**Demographics**			
Age, years	53.50 (42.00, 64.25)	59.00 (43.25, 67.25)	0.52
Male, n (%)	41 (64.06)	11 (68.75)	0.73
Lifestyle factors			
Smoking, n (%)	11 (17.46)	5 (31.25)	0.38
Alcohol consumption, n (%)	6 (9.52)	3 (18.75)	0.55
**Laboratory parameters**			
WBC, ×10⁹/L	6.59 ± 1.59	6.24 ± 2.36	0.48
HB, g/L	134.66 ± 19.58	103.44 ± 29.11	<0.001
PLT, ×10⁹/L	224.23 ± 65.64	166.88 ± 60.61	0.002
PT, s	10.90 ± 1.02	11.87 ± 1.92	0.07
APTT, s	28.95 ± 3.13	31.12 ± 6.57	0.22
D-D, mg/L	0.46 (0.21, 0.90)	1.00 (0.38, 1.62)	0.06
ALB, g/L	32.27 ± 8.59	32.23 ± 6.41	0.98
BUN, mmol/L	6.83 ± 2.46	15.29 ± 9.21	0.002
Cr, μmol/L	96.78 ± 58.75	303.62 ± 280.22	0.01
eGFR, mL/min/1.73 m ²	82.10 ± 28.77	43.82 ± 36.40	<0.001
24h UP, g/24h	3.50 ± 3.54	1.72 ± 1.30	0.002
TG, mmol/L	2.10 ± 1.39	2.19 ± 2.05	0.82
TC, mmol/L	5.46 (4.12, 6.66)	4.51 (3.59, 5.92)	0.24
**Comorbidities, n (%)**			
Diabetes mellitus	14 (22.22)	2 (12.50)	0.61
Hypertension	31 (49.21)	9 (56.25)	0.62
History of malignancy	7 (11.11)	2 (12.50)	1.00
AC/AP use	4 (6.35)	1 (6.25)	1.00
LLD use	9 (14.29)	1 (6.25)	0.66
Nephrotic syndrome	18 (28.12)	1 (6.25)	0.13

Data are presented as mean ± SD, median (Q1, Q3), or n (%). Bold values indicate statistical significance (*p* < 0.05).

Abbreviations: WBC, white blood cell count; HB, hemoglobin; PLT, platelet count; PT, prothrombin time; APTT, activated partial thromboplastin time; D-D, D-dimer; ALB, albumin; BUN, blood urea nitrogen; Cr, creatinine; eGFR, estimated glomerular filtration rate; 24h UP, 24-h urinary protein; TG, triglycerides; TC, total cholesterol; AC, anticoagulant agents; AP, antiplatelet agents; LLD, lipid-lowering drugs.

Regarding coagulation-related parameters, platelet count was significantly lower in cases than in controls (166.88 ± 60.61 × 10^9^/L vs. 224.23 ± 65.64 × 10^9^/L, *p* = 0.002). Prothrombin time (PT) tended to be prolonged in cases (11.87 ± 1.92 s vs. 10.90 ± 1.02 s, *p* = 0.07), and D‑dimer levels tended to be higher (1.00 mg/L vs. 0.46 mg/L, *p* = 0.06). Hemoglobin was significantly lower in cases (103.44 ± 29.11 g/L vs. 134.66 ± 19.58 g/L, *p* < 0.001). Albumin, triglycerides, and total cholesterol did not differ significantly between the two groups (all *p* > 0.05).

Regarding comorbidities, there were no significant differences between the two groups in the proportions of diabetes, hypertension, history of malignancy, use of anticoagulant or antiplatelet agents, use of lipid‑lowering drugs, or nephrotic syndrome (all *p* > 0.05).

The above differences in baseline characteristics—particularly in kidney function, coagulation parameters, and hemoglobin—highlight the need for adequate adjustment in multivariable analyses to identify the independent association between eGFR and severe bleeding.

### 3.2. Association between eGFR and severe post‑biopsy bleeding

Multivariable logistic regression was used to evaluate the association between eGFR and severe bleeding risk, with results detailed in Table 2. When eGFR was analyzed as a continuous variable, the crude model showed that each 1 mL/min/1.73 m ^2^ increase in eGFR was associated with a 4% reduction in severe bleeding risk (OR 0.96, 95% CI 0.95–0.98, *p* < 0.001). After adjustment for age and sex (Model I), the association remained stable (OR 0.96, 95% CI 0.95–0.98, *p* < 0.001). Further adjustment for smoking, alcohol consumption, diabetes, and hypertension (Model II) did not materially change the results (OR 0.96, 95% CI 0.94–0.98, *p* < 0.001). In Model III, which additionally adjusted for platelet count, prothrombin time, activated partial thromboplastin time, albumin, D‑dimer, triglycerides, total cholesterol, and 24‑hour urinary protein, each 1 mL/min/1.73 m^2^ increase in eGFR remained independently associated with a reduced bleeding risk (OR 0.96, 95% CI 0.93–0.98, *p* = 0.007).

**Table 2.** Association between eGFR and bleeding risk after renal biopsy.

Variable	Event, N (%)	Crude model		Model I		Model II		Model III	
		OR (95%CI)	P value	OR (95%CI)	P value	OR (95%CI)	P value	OR (95%CI)	P value
eGFR (mL/min/1.73 m²)	16/80 (20%)	0.96 (0.95 - 0.98)	<0.001	0.96 (0.95 - 0.98)	<0.001	0.96 (0.94 - 0.98)	<0.001	0.96 (0.93 - 0.98)	0.007
eGFR category									
<30	7/11 (83.6%)	1 (Reference)		1 (Reference)		1 (Reference)		1 (Reference)	
30–60	2/11 (18.2%)	0.13 (0.02 - 0.91)	0.04	0.13 (0.02 - 0.91)	0.04	0.14 (0.02 - 1.11)	0.06	0.47 (0.04 - 5.84)	0.56
≥60	7/58 (12.1%)	0.08 (0.02 - 0.34)	<0.001	0.08 (0.02 - 0.35)	<0.001	0.08 (0.02 - 0.37)	<0.001	0.09 (0.01 - 0.75)	0.03
*P* for trend			<0.001		<0.001		0.002		0.02

Abbreviations: OR, odds ratio; CI, confidence interval; PT, prothrombin time; APTT, activated partial thromboplastin time; PLT, platelets; ALB, albumin; D-D, D-dimer; TG, triglycerides; TC, total cholesterol; 24h UP, 24-h urinary protein.

Model descriptions.

Crude model: Unadjusted.

Model I: Adjusted for sex and age.

Model II:Adjusted for Model I + smoke, drink, history of diabetes and history of hypertension.

Model III: Adjusted for Model I + smoke, drink, history of diabetes and history of hypertension, PLT, PT, APTT, ALB, D-D, TG, TC, and 24h UP.

When eGFR was categorized into three levels according to KDIGO criteria (<30, 30–60, and ≥60 mL/min/1.73 m^2^), using the <30 mL/min/1.73 m^2^ group as the reference, the ≥60 mL/min/1.73 m^2^ group showed significantly lower bleeding risk across all models. In the fully adjusted Model III, the OR for the ≥60 mL/min/1.73 m^2^ group was 0.09 (95% CI 0.01–0.75, *p* = 0.03), while the OR for the 30–60 mL/min/1.73 m^2^ group was 0.47 (95% CI 0.04–5.84, *p* = 0.56). A significant dose–response relationship was observed across eGFR category (P for trend = 0.02).

**Figure 2.**
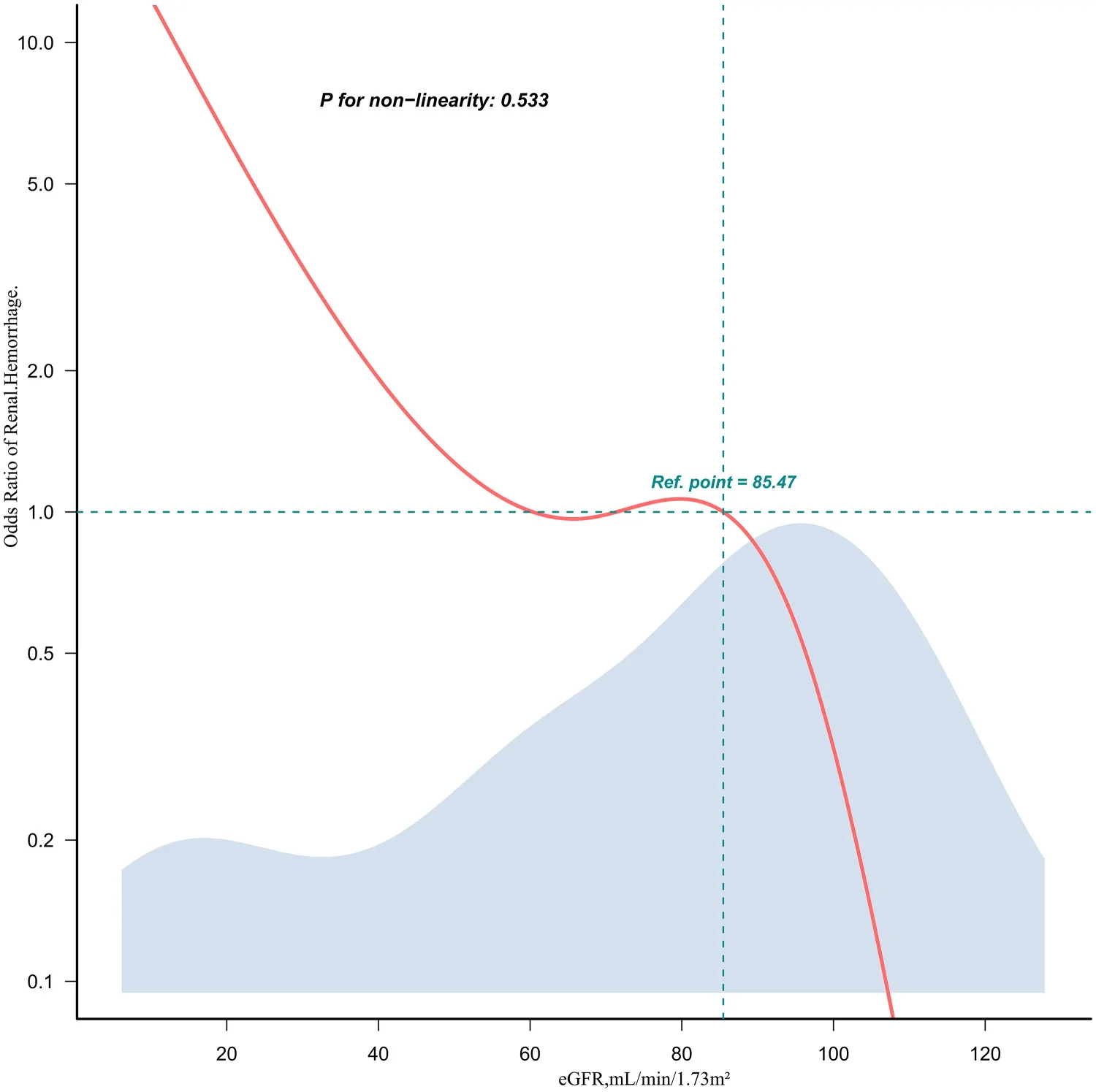
Restricted cubic spline analysis of eGFR and severe bleeding risk. Adjusted for age, sex, smoking, alcohol, diabetes, hypertension, PLT, PT, APTT, ALB, D-D, TG, TC, and 24h UP. P for non-linearity = 0.533, indicating a linear inverse association.

To further explore the dose–response relationship between eGFR and severe bleeding risk, restricted cubic spline regression was performed, which revealed a linear negative association (Figure 2).

Collectively, these findings suggest that lower eGFR may be an independent risk factor for severe bleeding requiring embolization after renal biopsy, with a linear dose–response relationship. However, given the limited sample size, these results should be interpreted as exploratory and require validation in larger cohorts.

### 3.3. Subgroup analyses

**Figure 3.**
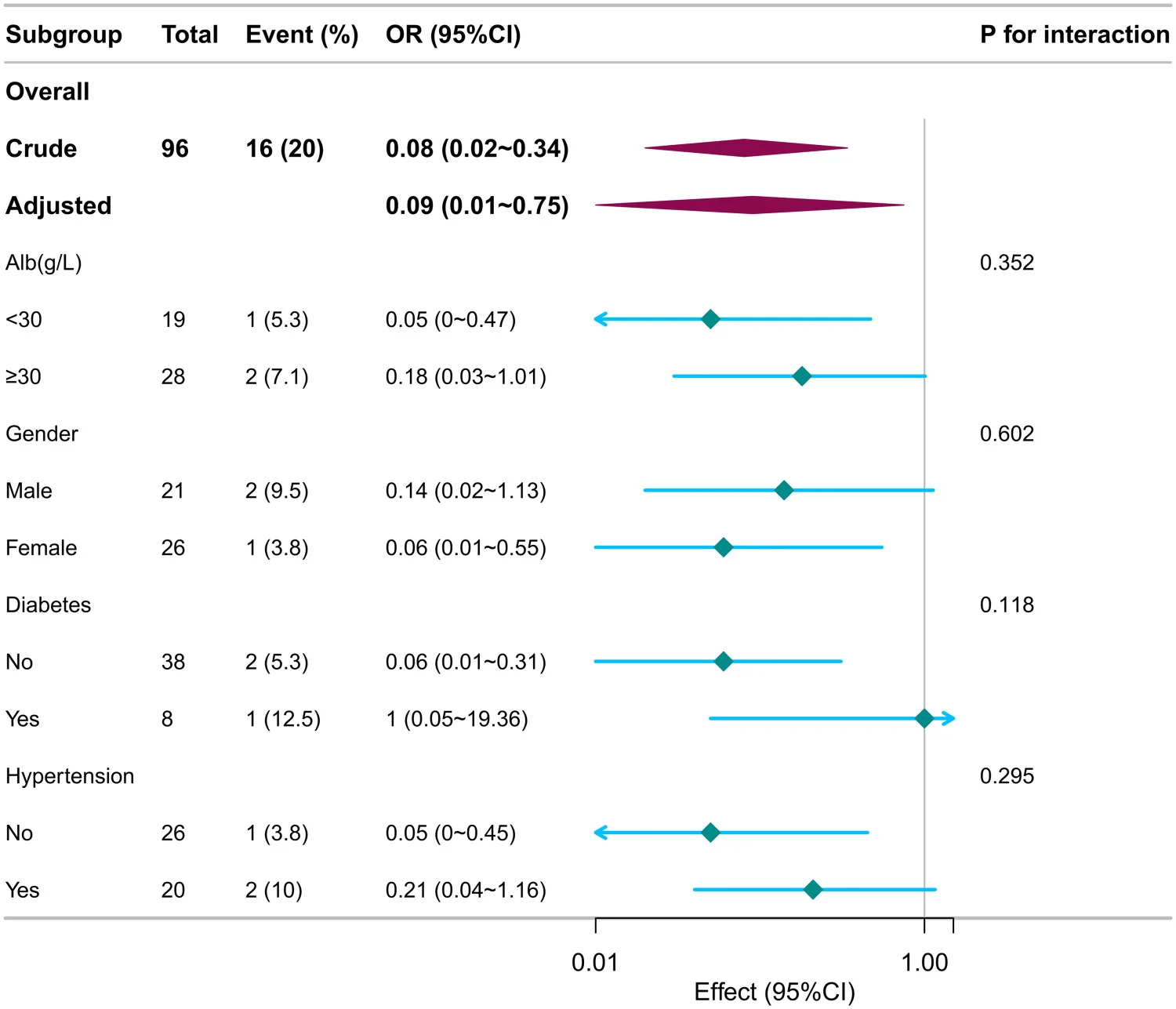
Subgroup analyses of the association between eGFR and severe bleeding. Adjusted for all covariates in Model III. eGFR ≥60 vs. <30 mL/min/1.73 m^2^. No significant interactions were observed (all *p* > 0.05).

To evaluate the stability of the association between eGFR and severe bleeding risk, subgroup analyses were performed according to prespecified stratification variables, including albumin level (<30 g/L vs. ≥30 g/L), sex, diabetes, and hypertension. The strength of the association between eGFR and severe bleeding risk in each subgroup was estimated using the fully adjusted Model III, and consistency across subgroups was assessed by interaction tests (Figure 3).

Overall, compared with eGFR <30 mL/min/1.73 m^2^, eGFR ≥60 mL/min/1.73 m^2^ consistently showed a protective trend against bleeding across all subgroups. In the albumin‑stratified analysis, both the albumin <30 g/L group (OR 0.05, 95% CI 0.00–0.47) and the albumin ≥30 g/L group (OR 0.18, 95% CI 0.03–1.01) showed a negative association, with a non‑significant interaction (P for interaction = 0.35), suggesting that albumin level did not significantly modify the association. In sex‑stratified analysis, both male (OR 0.14, 95% CI 0.02–1.13) and female (OR 0.06, 95% CI 0.01–0.55) subgroups demonstrated a negative association, with no significant interaction (P for interaction = 0.6). In diabetes‑stratified analysis, the non‑diabetes subgroup showed a significant negative association (OR 0.06, 95% CI 0.01–0.31), whereas the diabetes subgroup showed an estimate trending toward no association (OR 1.00, 95% CI 0.05–19.36); however, this subgroup had a small sample size (only 1 event) and a wide confidence interval. The interaction term was not significant (P for interaction = 0.12). In hypertension‑stratified analysis, both the non‑hypertension (OR 0.05, 95% CI 0.00–0.45) and hypertension (OR 0.21, 95% CI 0.04–1.16) subgroups showed protective trends, with no significant interaction (P for interaction = 0.30). Given the small sample sizes in some subgroups (e.g. only 1 event in the diabetes subgroup), these results should be interpreted with caution due to limited statistical power.

In summary, the inverse association between higher eGFR and lower severe bleeding risk was consistent across most subgroups, with no significant interactions detected, indicating that the association is stable across different clinical characteristics.

### 3.4. Sensitivity analyses

To further validate the robustness of the findings and mitigate the risk of overfitting due to multiple covariates, several sensitivity analyses were performed.

To assess the predictive value of eGFR for severe bleeding, a receiver operating characteristic (ROC) curve was constructed. The area under the curve (AUC) for eGFR was 0.80 (95% CI 0.68–0.92), indicating moderate predictive performance (Table S1). The optimal cut‑off value determined by the Youden index was 77.267 mL/min/1.73 m^2^ (Table S1 and Figure S1).

Based on this ROC‑derived cut‑off, eGFR was dichotomized into <77.267 and ≥77.267 mL/min/1.73 m^2^ for logistic regression analysis. As shown in Table S2, the unadjusted model showed that the ≥77.267 mL/min/1.73 m^2^ group had a significantly lower bleeding risk compared with the reference group (OR 0.10, 95% CI 0.03–0.41, *p* = 0.001). After full adjustment for age, sex, smoking, alcohol consumption, diabetes, hypertension, platelet count, prothrombin time, activated partial thromboplastin time, albumin, D‑dimer, triglycerides, and total cholesterol, the association remained robust (OR 0.05, 95% CI 0.01–0.41, *p* = 0.005). Consistent results were also observed in the complete‑case analysis (Table S3). These findings further support that lower eGFR is independently associated with an increased risk of severe bleeding.

Using variables selected by LASSO regression, a simplified multivariable logistic regression model was constructed to analyze the association between eGFR (three categories) and severe bleeding (Table S4 and Figure S2). In this LASSO‑selected model, using eGFR <30 mL/min/1.73 m^2^ as the reference, the eGFR ≥60 mL/min/1.73 m^2^ group showed a significantly reduced bleeding risk (OR 0.13, 95% CI 0.03–0.68, *p* = 0.02), while the eGFR 30–60 mL/min/1.73 m^2^ group showed a protective trend that did not reach statistical significance (OR 0.44, 95% CI 0.05–4.15, *p* = 0.48). A significant dose–response relationship was observed (P for trend = 0.01).

These sensitivity analyses yielded results highly consistent with the primary analysis (Model III), further confirming that lower eGFR is an independent risk factor for severe bleeding and that the variable selection process did not alter the core conclusions, indicating good robustness of the findings.

## 4. Discussion

This matched case–control study demonstrates that lower estimated glomerular filtration rate (eGFR) is independently associated with an increased risk of severe post‑biopsy bleeding requiring selective renal artery embolization. While previous studies have identified reduced eGFR as an independent predictor of post‑biopsy bleeding [4], the present study extends this association to the most severe bleeding phenotype—hemorrhage requiring vascular intervention—and employs a matched case–control design to optimize analysis of this rare outcome.

The incidence of severe bleeding in our center (0.37%, 16/4,321) aligns closely with reported rates of intervention‑requiring hemorrhage. In a systematic review of 118,064 renal biopsies by Poggio et al. [2], the transfusion rate was 1.6% and the vascular intervention rate was 0.3%. Halimi et al. [10], using a French nationwide cohort of 52,138 patients, reported a vascular intervention rate of 0.4%. Lees et al. [13] found similar rates in 2,563 native kidney biopsies at a UK center, with 0.4% (9/2,563) requiring embolization and 1.8% (46/2,563) requiring transfusion. The embolization rate in our study (0.37%) is consistent with these large‑scale reports, providing complementary epidemiological data focused specifically on the most severe bleeding endpoint.

Impaired kidney function is a clinically relevant risk factor for post‑biopsy bleeding, and multiple independent studies have consistently confirmed an inverse association between eGFR and bleeding risk. Halimi et al. [10] demonstrated that each 10 mL/min/1.73 m^2^ decrease in eGFR was associated with an 18% increase in major bleeding events. Lees et al. [13] identified reduced eGFR as an independent risk factor for severe bleeding requiring embolization. Fatthy et al. [14], in a large cohort of 1,198 patients (including both native and transplant kidneys), found that eGFR <30 mL/min/1.73 m^2^ was a strong independent predictor of major complications after native kidney biopsy (OR 9.7, 95% CI 3.4–18.2), with a similar effect observed in transplant kidneys (OR 11.3, 95% CI 3.5–16.8). Lim et al. [15] developed a risk prediction model incorporating age ≥62 years, hemoglobin <10 g/dL, and platelet count ≤216 × 10^9^/L in a high‑risk population (serum creatinine ≥2 mg/dL), demonstrating good predictive performance in patients with a mean eGFR of 18.8 mL/min/1.73 m^2^. A 2025 systematic review by Pacini et al. [16] (2,470 patients) further highlighted the clinical relevance of bleeding risk management in patients with kidney impairment, showing that desmopressin may reduce bleeding risk in high‑risk populations (eGFR <60 mL/min/1.73 m^2^) (observational studies: RR 0.52), suggesting that eGFR not only predicts risk but may also guide preventive interventions. By using a matched case–control design to analyze a rare outcome and focusing specifically on the most severe bleeding phenotype requiring vascular intervention, the present study strengthens the evidence base linking eGFR to severe bleeding.

Notably, some studies have reported findings divergent from ours. Xu et al. [6] found no significant association between eGFR and severe bleeding (defined as transfusion or hemoglobin drop ≥1 g/dL) in a retrospective study of 477 patients (OR 0.99, 95% CI 0.96–1.02). The Boston Kidney Biopsy Cohort study [5] found that although eGFR <30 mL/min/1.73 m^2^ was associated with transfusion risk in univariate analysis (OR 3.89, 95% CI 1.76–8.58), this association lost statistical significance after adjustment for hemoglobin (OR 1.55, 95% CI 0.65–3.70, *p* = 0.33). Several methodological differences may explain these discrepancies. First, outcome definitions differ substantially: those studies defined severe bleeding as transfusion or vascular intervention, whereas we focused specifically on the most severe phenotype—bleeding requiring embolization. Transfusion decisions are subjective and influenced by baseline hemoglobin levels, whereas embolization is guided by objective imaging findings, making outcome ascertainment more definitive. The underlying pathophysiology may also differ: transfusion‑requiring bleeding largely reflects hemoglobin decline, whereas embolization‑requiring bleeding depends on persistent active bleeding and vascular injury. Second, confounding control strategies differed: those studies adjusted primarily for hemoglobin, whereas we controlled for age and sex through 1:4 matching and further adjusted for platelet count, coagulation parameters, and other potential confounders selected *via* LASSO regression, enabling more precise estimation of the independent effect of eGFR. These differences underscore the critical importance of precise outcome definition and adequate confounding control in bleeding studies, and suggest that the relationship between eGFR and bleeding risk may vary across bleeding severity.

The relative contributions of patient factors versus procedural factors to bleeding risk warrant consideration. Spanuchart et al. [17] showed that after adjusting for patient factors (including kidney function, platelet count, and coagulation parameters), technical variables such as biopsy angle and needle depth were no longer statistically significant, suggesting that patient characteristics—including eGFR—may influence bleeding risk more than procedural factors. Monahan et al. [18], in a large retrospective study of 2,204 renal biopsies, found that although eGFR <60 mL/min/1.73 m^2^ was significantly associated with bleeding risk (*p* = 0.025), the overall major complication rate was only 1.64% (37/2,204). Even in patients with impaired kidney function, standardized techniques (18 G needle, ultrasound guidance) enabled safe biopsy performance—emphasizing that risk stratification should guide enhanced post‑biopsy monitoring rather than precluding biopsy in patients with reduced eGFR. The systematic review by Pacini et al. [16] further demonstrated that desmopressin prophylaxis in high‑risk patients may reduce severe bleeding risk by approximately 50%, supporting eGFR not only as a risk marker but also as a potential guide for preventive interventions. Collectively, these findings suggest that reduced eGFR should not be considered a contraindication to renal biopsy, but rather a signal for intensified preoperative assessment and post‑procedural monitoring.

The mechanisms by which kidney impairment increases bleeding risk remain incompletely elucidated. Based on available evidence, we propose the following hypotheses: (1) Platelet dysfunction: in the uremic environment, impaired platelet function—including abnormalities in adhesion, aggregation, and release—is a central mechanism underlying bleeding tendency. Uremic toxins can inhibit expression and conformational changes of platelet membrane glycoprotein receptors (e.g. GPIb and GPIIb/IIIa), and may activate adenylate cyclase, increase cAMP production, and impair calcium mobilization, thereby interfering with platelet activation [11,12]. (2) Endothelial and coagulation abnormalities: accumulated metabolic products such as urea and guanidine compounds may damage vascular endothelium, increase vascular fragility, and impair repair capacity after injury. Concurrently, hepatic synthesis of coagulation factors may be compromised by malnutrition or uremic status [12]. (3) Anemia and tissue hypoxia: chronic kidney disease is often accompanied by anemia; reduced hemoglobin levels lead to inadequate tissue oxygen delivery, potentially compromising local hemostatic capacity and vasoconstrictive responses. In our study, the case group exhibited significantly lower platelet counts and hemoglobin levels, consistent with these mechanisms. Notably, the association between lower eGFR and bleeding risk persisted after adjustment for platelet count and coagulation parameters, suggesting that eGFR may capture additional pathophysiological processes beyond these measured factors, such as the cumulative burden of uremic toxins that are difficult to fully adjust for using routine laboratory tests.

In univariate analyses, we identified several other factors associated with bleeding risk. Compared with controls, cases had significantly lower platelet counts, higher blood urea nitrogen and serum creatinine levels, and lower eGFR—findings consistent with the existing literature. Fontana et al. [19], in a single‑center observational study of 819 biopsies (285 native, 534 transplant), found that low eGFR (particularly <30 mL/min/1.73 m^2^) was significantly associated with bleeding complications, with an adjusted OR of 3.57 (95% CI 1.76–7.23, *p* < 0.001). Asad et al. [20], in a prospective study of high‑risk patients with eGFR <30 mL/min/1.73 m^2^, reported a major complication rate of 5.6% and a perirenal hematoma detection rate of 37.3%, with significantly higher rates of hematoma ≥2 cm in patients with eGFR <15 mL/min/1.73 m^2^ (29.2% vs. 13.0%, *p* = 0.032). Jung et al. [21], in a 7‑year retrospective study of 627 patients, found that obesity was independently associated with reduced complication risk (adjusted OR 0.5, 95% CI 0.34–0.74, *p* < 0.001), while low eGFR remained an independent predictor of increased bleeding risk—further supporting the important influence of patient characteristics, including kidney function and BMI, on bleeding risk. Zheng et al. [3], in a nested case–control study, identified D‑dimer (OR 1.27, 95% CI 1.02–1.57) and eGFR (OR 0.90, 95% CI 0.84–0.96) as independent predictors of severe bleeding, with excellent model performance (AUC = 0.91). Collectively, these studies underscore the multifactorial nature of post‑biopsy bleeding risk and highlight the need for comprehensive preoperative evaluation.

ROC curve analysis showed that eGFR alone had moderate predictive value for severe bleeding (AUC = 0.80, 95% CI 0.68–0.92). The optimal cut‑off value determined by the Youden index was 77.267 mL/min/1.73 m^2^, which could serve as a simple bedside tool: patients with eGFR ≤77.267 mL/min/1.73 m^2^ warrant heightened vigilance and intensified post‑biopsy monitoring. However, as demonstrated by Zheng et al. [3] (AUC reached 0.91 after incorporating clinical and ultrasonographic features) and Li et al. [4] (AUC improved from 0.683 to 0.786 after adding immediate post‑biopsy ultrasound data, and to 0.867 after further incorporating 6‑hour hematoma size), integrating eGFR with other clinical and ultrasound parameters can substantially enhance predictive performance. Therefore, while eGFR alone provides some value for risk stratification, a multidimensional approach enables more accurate identification of high‑risk individuals.

Several limitations should be acknowledged. First, the single‑center retrospective design and small sample size—particularly only 16 cases—may introduce selection and information biases, reduce statistical power, and compromise the stability of subgroup analyses. In addition, by defining controls as patients with no bleeding on post‑biopsy ultrasound, we excluded those with minor or moderate hematomas. This design compares severe bleeding against the absence of any bleeding, rather than against mild-to-moderate bleeding—a comparison that may influence effect estimates. However, this approach was chosen to maximize contrast between the two groups for the most clinically meaningful outcome, and the findings should be interpreted in this context. Second, the absence of bleeding on post‑biopsy ultrasound in the control group does not exclude the presence of small, clinically silent hematomas. Ultrasound is less sensitive than CT or MRI, which can detect hematomas in a much higher proportion of patients. However, our primary endpoint was embolization-requiring bleeding—an objective, intervention-driven outcome rather than a radiological finding. Thus, while ultrasound may have underestimated the overall incidence of any post-biopsy bleeding, this limitation does not affect the validity of our findings regarding the association between eGFR and severe, clinically meaningful bleeding events.Third, although we controlled for known confounders through multivariable adjustment, LASSO variable selection, and 1:4 matching, residual confounding from unmeasured variables cannot be excluded. For example, Zhan et al. [22] demonstrated that 16 G needles carry higher bleeding risk than 18 G needles, suggesting needle gauge is a potential confounder not captured in our study; Wang et al. [23] highlighted that accessory renal artery injury is an important mechanism of severe bleeding, suggesting potential value of preoperative vascular imaging. Additionally, variables such as timing of antiplatelet/anticoagulant discontinuation, preoperative blood pressure levels, and number of needle passes were not included in the analysis. Of note, LASSO regression was specifically applied to mitigate the risk of overfitting given the limited number of outcome events (*n* = 16), and the selected variables were entered into a simplified multivariable model to obtain final estimates. Fourth, the optimal eGFR cut‑off value identified in this study (77.267 mL/min/1.73 m^2^) lacks external validation and requires confirmation in independent cohorts. Moreover, the low sensitivity (29%) suggests that using eGFR alone may miss some high‑risk patients; therefore, clinical application should combine eGFR with other indicators. Fifth, eGFR was calculated using the CKD-EPI equation rather than more precise methods such as cystatin C or radionuclide renography, which may introduce measurement bias. Sixth, we did not incorporate pathological findings—such as specific diagnoses, degree of interstitial fibrosis, or the presence of vasculitis—into our bleeding risk analysis. While these factors may influence bleeding propensity [24], our study was deliberately designed to focus on pre‑biopsy variables that are routinely available to clinicians for point‑of‑care risk assessment. Future investigations with larger cohorts are needed to determine whether pathological characteristics modify the association between eGFR and post‑biopsy bleeding. Finally, this study was conducted in a single Chinese population, and extrapolation to other ethnic groups should be made with caution.

## 5. Conclusions

Lower eGFR may be an independent risk factor for severe bleeding requiring selective renal artery embolization after percutaneous renal biopsy, with a linear dose–response relationship. However, given the exploratory nature of this study due to its limited sample size, these findings should be interpreted with caution. If confirmed in larger cohorts, eGFR may have utility in preoperative bleeding risk assessment, and patients with reduced kidney function may warrant more intensive post-biopsy monitoring. Future large-scale, multicenter prospective studies with external validation are needed to confirm these results and to develop robust multivariable prediction models.

## Supplementary Material

TableS3 eGFR.docx

TableS4 eGFR.docx

TableS1 eGFR.docx

FigureS1.tif

TableS2 eGFR.docx

Figure S2.tif

## Data Availability

The dataset used during this study available from the corresponding author on reasonable request.
