# Supplementary material for: Lower eGFR is independently associated with embolization‑requiring severe bleeding after percutaneous renal biopsy: a matched case–control study with dose–respons e analysis
Source: Ren Fail. 2026 Sep 3;48(1):2714663. doi: 10.1080/0886022X.2026.2714663 (PMC13552286; doi:10.1080/0886022X.2026.2714663)
Supplement: Supplementary file 1 [file IRNF_A_2714663_SM7713.docx]

****Table S2. Association between dichotomized eGFR and severe post-biopsy bleeding****

| Variable | n.total | Events (%) | Crude OR (95% CI) | Crude P-value | Adjusted OR (95% CI) | Adjusted P-value |
| --- | --- | --- | --- | --- | --- | --- |
| eGFR<77.267 | 33 | 13/33 (39.4%) | 1.00 (Reference) |  | 1.00 (Reference) |  |
| eGFR≥77.267 | 47 | 3/47 (6.4%) | 0.10 (0.03-0.41) | 0.001 | 0.05 (0.01-0.41) | 0.005 |

Abbreviations: eGFR, estimated glomerular filtration rate; OR, odds ratio; CI, confidence interval; PLT, platelets; PT, prothrombin time; APTT, activated partial thromboplastin time; ALB, albumin; D-D, D-dimer; TG, triglycerides; TC, total cholesterol.

Model descriptions:
Crude model: Unadjusted.
Adjusted model: Adjusted for age, sex, smoking, alcohol, diabetes, hypertension, PLT, PT, APTT, ALB, D-D, TG, and TC.
