# Supplementary material for: Lower eGFR is independently associated with embolization‑requiring severe bleeding after percutaneous renal biopsy: a matched case–control study with dose–respons e analysis
Source: Ren Fail. 2026 Sep 3;48(1):2714663. doi: 10.1080/0886022X.2026.2714663 (PMC13552286; doi:10.1080/0886022X.2026.2714663)
Supplement: Supplementary file 2 [file IRNF_A_2714663_SM7715.docx]

Table S1. Predictive performance of eGFR for severe bleeding after renal biopsy.

| Parameter | AUC  (95% CI) | Accuracy  (95% CI) | PPV  (95% CI) | NPV  (95% CI) | Cut-off value |
| --- | --- | --- | --- | --- | --- |
| eGFR | 0.80 (0.68-0.92) | 0.29 (0.19-0.40) | 0.61 (0.44 - 0.77) | 0.06 (0.00 - 0.13) | 77.267 |

Abbreviations: AUC, area under the curve; CI, confidence interval; PPV, positive predictive value; NPV, negative predictive value;eGFR, estimated glomerular filtration rate.
