# Supplementary material for: Lower eGFR is independently associated with embolization‑requiring severe bleeding after percutaneous renal biopsy: a matched case–control study with dose–respons e analysis
Source: Ren Fail. 2026 Sep 3;48(1):2714663. doi: 10.1080/0886022X.2026.2714663 (PMC13552286; doi:10.1080/0886022X.2026.2714663)
Supplement: Supplementary file 3 [file IRNF_A_2714663_SM7716.docx]

**Table S4. Association between eGFR category and severe post-biopsy bleeding after LASSO variable selection**

| **Variable** | **Event，N(%)** | **Crude model** | | **Model** I | |
| --- | --- | --- | --- | --- | --- |
|  |  | **OR (95%CI)** | **P value** | **OR (95%CI)** | **P value** |
| eGFR category |  |  |  |  |  |
| <30 | 7/11 (83.6%) | 1 (Reference) |  | 1 (Reference) |  |
| 30-60 | 2/11 (18.2%) | 0.13 (0.02~0.91) | 0.04 | 0.44 (0.05~4.15) | 0.48 |
| ≥60 | 7/58 (12.1%) | 0.08 (0.02~0.34) | <0.001 | 0.13 (0.03~0.68) | 0.02 |
| *P* for trend |  |  | <0.001 |  | 0.01 |

Abbreviations: eGFR, estimated glomerular filtration rate; OR, odds ratio; CI, confidence interval; PT, prothrombin time; APTT, activated partial thromboplastin time; PLT, platelets.

Model descriptions:

Crude model: Unadjusted.

Model I: Adjusted for PT, APTT, PLT, 24h UP.
