# Supplementary material for: Lower eGFR is independently associated with embolization‑requiring severe bleeding after percutaneous renal biopsy: a matched case–control study with dose–respons e analysis
Source: Ren Fail. 2026 Sep 3;48(1):2714663. doi: 10.1080/0886022X.2026.2714663 (PMC13552286; doi:10.1080/0886022X.2026.2714663)
Supplement: Supplementary file 4 [file IRNF_A_2714663_SM7717.docx]

**Table S3. Sensitivity Analysis: Association Between eGFR and Severe Post-Biopsy Bleeding in Complete-Case Analysis**

| **Variable** | **Event,N(%)** | **Crude model** | | **Model I** | | **Model Ⅱ** | | **Model Ⅲ** | |
| --- | --- | --- | --- | --- | --- | --- | --- | --- | --- |
|  |  | **OR(95%CI)** | **P value** | **OR(95%CI)** | **P value** | **OR(95%CI)** | **P value** | **OR(95%CI)** | **P value** |
| eGFR category |  |  |  |  |  |  |  |  |  |
| <30 | 7/11 (83.6%) | 1 (Reference) |  | 1 (Reference) |  | 1 (Reference) |  | 1 (Reference) |  |
| 30-60 | 2/11 (18.2%) | 0.13 (0.02~0.91) | 0.04 | 0.13 (0.02~0.91) | 0.04 | 0.15 (0.02~1.11) | 0.06 | 0.52 (0.04~7.07) | 0.62 |
| ≥60 | 7/58 (12.1%) | 0.08 (0.02~0.34) | 0.001 | 0.08 (0.02~0.35) | 0.001 | 0.08 (0.02~0.37) | 0.001 | 0.10 (0.01~0.80) | 0.03 |
| *P* for trend | 16/80 (20%) |  | 0.001 |  | 0.001 |  | 0.002 |  | 0.02 |

Abbreviations: eGFR, estimated glomerular filtration rate; OR, odds ratio; CI, confidence interval; PLT, platelets; PT, prothrombin time; APTT, activated partial thromboplastin time; ALB, albumin; D-D, D-dimer; TG, triglycerides; TC, total cholesterol; 24h UP, 24-hour urinary protein.

Model descriptions:

Crude model: unadjusted.

Model I: adjusted for age and sex.

Model II: adjusted for Model I plus smoking, alcohol consumption, diabetes mellitus, and hypertension.

Model III: adjusted for Model II plus PLT, PT, APTT, ALB, D-D, TG, TC, and 24h UP.
